# Protein Phosphorylation: An Essential Role in Shoot Apical Meristem Homeostasis

**DOI:** 10.3390/plants15132033

**Published:** 2026-06-30

**Authors:** Cuicui Qi, Qianqian Qin, Suiwen Hou

**Affiliations:** Gansu Province Key Laboratory of Gene Editing for Breeding, Ministry of Education on Key Laboratory of Cell Activities and Stress Adaptations, Gansu Province Ministry of Education, School of Life Sciences, Lanzhou University, Lanzhou 730000, China; qicc21@lzu.edu.cn (C.Q.);

**Keywords:** shoot apical meristem, phosphorylation, CLAVATA, WUSCHEL, receptor-like kinases

## Abstract

The shoot apical meristem (SAM) serves as the cellular source of aboveground plant development and is precisely regulated by a complex interplay of genetic, hormonal, and environmental factors. Central to this regulation is the CLAVATA3 (CLV3)–WUSCHEL (WUS) negative feedback loop, which maintains SAM homeostasis by balancing stem cell proliferation and differentiation. Among the diverse regulatory mechanisms, reversible protein phosphorylation, which is mediated by protein kinases and phosphatases, has emerged as a key posttranslational modification that integrates internal and external signals to modulate SAM activity. This review summarizes recent advances in understanding the roles of kinases and phosphatases in SAM maintenance, with a particular focus on phosphorylation-mediated control of the CLV3–WUS pathway and associated signaling networks. By synthesizing these molecular insights, we aim to provide a comprehensive reference for deciphering the regulatory mechanisms underlying SAM homeostasis. A deeper understanding of SAM regulation not only advances fundamental knowledge of plant developmental biology but also holds significant potential for improving crop architecture and agricultural productivity.

## 1. Introduction

Unlike in animals, whose organs form during embryogenesis, most organs of plants form throughout their lifecycle [1]. The growth of plants is sustained by two primary meristems, the shoot apical meristem (SAM) and the root apical meristem (RAM), which govern aboveground and root development, respectively. The continuous production of aboveground organs originates from the stem cell population within the SAM [2,3]. Stem cells maintain themselves through division and differentiate to replenish the surrounding tissue [4,5]. During the vegetative growth stage, the SAM continuously undergoes cell division, maintaining the stability of its own stem cell population while providing cells for the initiation of leaf primordia [6]. When plants transition from vegetative growth to reproductive growth, SAM transforms into the inflorescence meristem (IM), which then generates the floral meristem (FM), ultimately forming floral organs [7,8]. In maize and tomato, the homeostatic regulation of SAM is intimately associated with yield [9,10,11,12,13]. Therefore, understanding the regulatory mechanism of the SAM is highly important for the study of plant development and agricultural production.

Protein phosphorylation, one of the most important posttranslational modifications in plants, is involved in the regulation of various aspects of plant growth and development. Phosphorylation affects protein activity and stability and alters intracellular localization and protein-protein interactions [14,15,16,17]. Protein phosphorylation is catalyzed by protein kinases, whereas dephosphorylation is mediated by protein phosphatases. These two processes antagonistically regulate the phosphorylation state of proteins, thereby enabling them to exert their functions precisely [18]. Recent studies have shown that phosphorylation plays a crucial role in regulating the homeostasis of SAM [19]. This review summarizes the role of phosphorylation in the regulation of SAM homeostasis, focusing on the key protein kinases, phosphatases, and phosphorylation events involved. This study aims to serve as a reference for a comprehensive understanding of this regulatory mechanism.

## 2. Shoot Apical Meristem Structure

**Figure 1:**
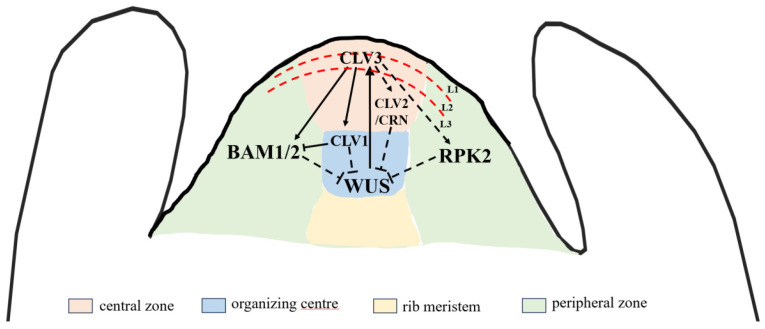
The CLV3-WUS negative feedback regulatory pathway in the SAM. The Arabidopsis SAM is organized into four distinct zones: the CZ, highlighted in pink, located at the summit of the SAM; the PZ, outlined in green, which surrounds the CZ; the RM, shown in yellow, situated beneath the CZ; and the OC, depicted in blue, positioned between the CZ and the RM. Within the CZ, three layers of stem cells—L1, L2, and L3—are demarcated by red dashed lines. The CZ serves as the source of the CLV3 ligand, which is secreted from stem cells and perceived by a suite of plasma membrane-localized receptor complexes, including CLV1, CLV2/CRN heterodimers, RPK2, and BAM1/2 receptors, as indicated in the periphery of the CZ. Upon ligand perception, the signal is transduced to the nucleus through an as-yet-uncharacterized mechanism, leading to the repression of *WUS* expression in the OC. This negative feedback loop, operating between the CZ and the OC, is fundamental for restricting stem cell proliferation and maintaining SAM homeostasis. The solid black lines represent direct interactions, while the dashed black lines indicate that direct interactions have not been reported. Arrows indicate promotion/promoting effects, and T-bars indicate inhibition/inhibiting effects.

In Arabidopsis, the SAM is a hemispherical structure located at the top of the plant. It can be divided into four regions based on its function: the central zone (CZ), the organizing center (OC), the peripheral zone (PZ), and the rib meristem (RM) (Figure 1). The CZ is located at the center and top of the SAM and harbors pluripotent stem cells, which are the source of all above-ground tissues of the plant except the cotyledons. Some stem cells can move from the CZ to the PZ and rapidly divide to form organ primordia. The OC, situated beneath the CZ, is composed of quiescent cells that maintain the stem cell niche of the CZ. The RM is located at the bottom of the SAM and provides mainly primary cells for stem development [2,6,20,21,22]. On the basis of cell arrangement, the SAM is organized into three concentric layers (L1, L2, and L3). The stem cells that constitute these layers are located primarily in the CZ (Figure 1). Both the L1 layer and the L2 layer are single-layered cells that generate epidermal tissue and subepidermal tissue through apical division, respectively. The L3 layer is a multilayer cell that provides initiating cells for vascular tissues through multipolar division [23,24].

## 3. CLV3-WUS Feedback Loop: A Conserved Central Module for Shoot Apical Meristem Homeostasis

Over the past few decades, numerous regulatory components and signaling networks that influence meristem homeostasis have been identified, primarily including ligand–receptor and hormone pathways and transcription factors [23]. Among these, the negative feedback regulatory loop composed of CLV3-WUS plays a central role [25,26,27] (Figure 1).

### 3.1. CLV3-WUS Feedback Loop and Its Role in Shoot Apical Meristem Homeostasis

CLV3 is a secreted small peptide belonging to the CLV3/EMBRYO SURROUNDING REGION (ESR)-related (CLE) family and serves as a key molecular marker for stem cells in the SAM [28]. It is predominantly expressed in stem cells within the CZ of the SAM. The loss of function of CLV3 leads to an increased number of stem cells and enlargement of the SAM [25]. Compared with the wild type, the *clv3* mutant produces more rosette leaves during vegetative growth, and upon transitioning to reproduction, it develops enlarged inflorescence meristems in increased numbers, leading to the formation of club-shaped siliques that often bear extra valves [29]. Studies have shown that CLV3 can be perceived by three groups of receptors, namely, CLV1, CLV2/CORYNE (CRN), and RECEPTOR-LIKE PROTEIN KINASE 2 (RPK2) [29,30,31,32,33] (Figure 1). CLV3-INSENSITIVE KINASES (CIKs) act as coreceptors for CLV1, CLV2/CRN, and RPK2; transmit the signal of CLV3; and regulate the homeostasis of the SAM [34].

The key role of the CLV3 signaling pathway in meristem homeostasis is to restrict the expression of the homeodomain transcription factor *WUS*, which can inhibit the differentiation of CZ cells and maintain stem cell fate [26]. *WUS* is predominantly expressed in the OC of the SAM. In *wus* mutants, excessive differentiation of stem cells leads to premature termination of the SAM, resulting in a failure to produce leaves continuously, inability to bolt normally, and a lack of seed formation [35,36,37,38]. WUS moves to the CZ through plasmodesmata and regulates *CLV3* expression in a dose-dependent manner through binding to its promoter. At low concentrations, WUS exists as monomers or heterodimers that recruit coactivators to promote transcription, whereas at high concentrations, it forms repressive homodimers that lack this recruitment ability [39,40,41,42,43].

HAIRY MERISTEMS (HAMs) constitute a class of GRAS domain transcription factors that play crucial roles in maintaining plant meristems [44]. *HAM1/2* are widely expressed in the SAM, except for the L1–L2 layers, including in the PZ, OC, and RM. This expression pattern overlaps with that of the *WUS* gene and is complementary to the expression pattern of *CLV3* [45]. Research has shown that HAMs are pivotal for structuring the stem cell niche. The phenotypes of the *ham1/2/3/4* quadruple mutant are similar to those of the *wus* mutant, including premature differentiation of stem cells and early termination of the SAM [44,46]. At the molecular level, HAMs in the OC repress *CLV3* expression. Spatially, this inhibition confines *CLV3* activation to the CZ, where HAMs are absent. Thus, HAMs govern the spatial organization of stem cell activity through their cell type-specific expression [47].

### 3.2. CLV3-WUS Feedback Loop for Yield Improvement in Crops

The CLV3-WUS negative feedback regulatory loop is highly conserved in higher plants [48]. ZmFCP1, ZmCLE7, and ZmCLE14 from the maize CLE family, as homologs of AtCLV3, also influence the size of the SAM [49]. Maize has two WUS homologs, ZmWUS1 and ZmWUS2; *ZmWUS1* is expressed in the organizing center of the SAM, and its expression domain expands in *FEA3* (the CLV1 homolog) mutants, whereas *ZmWUS2* is expressed in the peripheral zone of the SAM and in leaf primordia [49,50]. The RAMOSA1 ENHANCER LOCUS2 (REL2) family of transcriptional corepressors in maize, including ZmREL2, ZmRELK1, ZmRELK2, and ZmRELK3, plays key roles in meristem development and reproductive success. They not only coordinate meristem development but also regulate yield-related traits [9]. The gene *ZmEREB14*, which was recently identified via single-cell RNA sequencing (scRNA-seq) technology, is also a key regulator of shoot apex development and yield formation [10].

In addition to maize, the CLV-WUS negative feedback regulatory loop is conserved in tomato. SlCLV3, the homolog of CLV3 in tomato, shares a conserved function with CLV3, and its mutation leads to the enlargement of the SAM in tomato. SlWUS, the homolog of WUS in tomato, is also expressed in the organizing center of the SAM [51]. Notably, the CLV3-WUS signaling pathway serves as a central regulator not only for meristem maintenance but also for key yield-related traits in tomato. A primary determinant of final fruit size is the carpel and locule number, which is established during the floral meristem (FM) stage [52]. SlCLV3 and SlWUS collaboratively regulate stem cell homeostasis through a negative feedback loop. Weakened CLV3 signaling—such as through promoter mutations at the *fas* locus—or sustained activation of *WUS* expression—as seen with mutations at the *lc* locus affecting its downstream CArG-box element—both release the inhibition of stem cell proliferation. This leads to an enlargement of the floral meristem and ultimately results in larger fruits and increased yield [53,54]. The ethylene response factor ENHANCER OF NON-SPECIFIC CELL PROLIFERATION 1 (ENO) has been identified as a flower-specific negative regulator that restricts stem cell proliferation by directly repressing *SIWUS* expression [55].

## 4. Phosphorylation of Shoot Apical Meristem Homeostasis

Protein phosphorylation is among the most common and prominent posttranslational modifications (PTMs) and is dynamically and antagonistically regulated by protein kinases and phosphatases [56]. This modification also plays a central role in the CLV3 signaling pathway, where it mediates the transmission of the CLV3 peptide signal from the extracellular space to the nucleus. Specifically, the CLV3 peptide is perceived by the extracellular domains of membrane-localized receptor kinases, thereby initiating intracellular signaling cascades largely driven by phosphorylation events to regulate *WUS* expression.

### 4.1. CLV3 Signal Transmission in Shoot Apical Meristem Homeostasis

**Figure 2:**
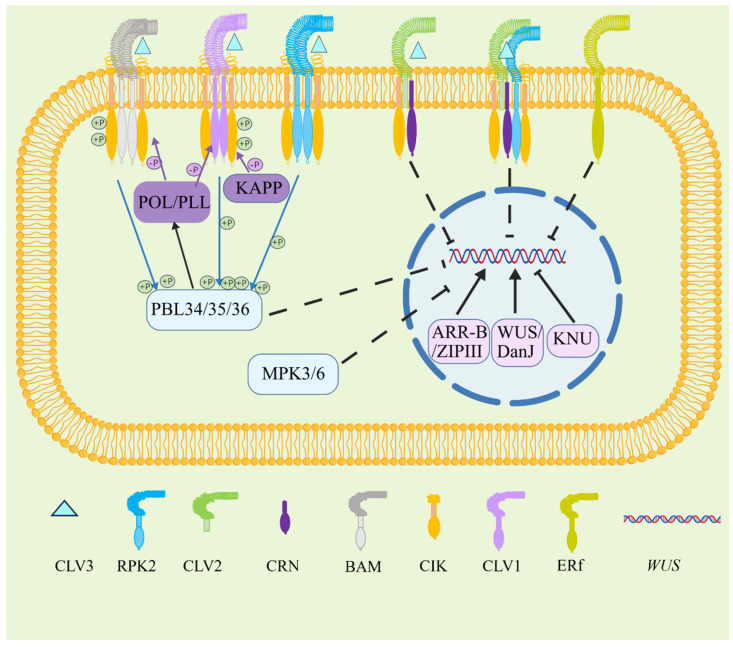
The CLV-centered plasma membrane signaling network regulates shoot apical meristem homeostasis. The plasma membrane harbors a diverse array of receptor kinases and receptor complexes, including CLV1, RPK2, the CLV2/CRN heterodimer, BAM receptors, ERf, and CIK co-receptors. Upon perception of the CLV3 ligand, these receptors initiate downstream signaling cascades. The cytoplasmic kinases PBL34, PBL35, and PBL36 physically interact with CLV1, BAM1/3, and CIKs, and are phosphorylated by CLV1 and BAM1. Concurrently, signaling components such as POL, PLL1, and KAPP mediate signal transduction from CLV receptors and function to dephosphorylate LRR-RLKs, thereby fine-tuning and attenuating CLV signaling output. In the nucleus, transcriptional activation of *WUS* is driven by two distinct complexes: the cytokinin signaling-activated type-B ARABIDOPSIS RESPONSE REGULATOR (ARR-B) transcription factor in complex with class III HOMEODOMAIN-LEUCINE ZIPPER (HD-ZIPIII) proteins, and the WUS–DnaJ domain-containing protein complex. In contrast, the transcription factor KNUCKLES (KNU) represses *WUS* transcription/translation. Green “+P” denotes enhanced phosphorylation, whereas purple “–P” indicates reduced phosphorylation. This integrated signaling network, spanning from plasma membrane perception to nuclear transcriptional regulation, collectively ensures the precise maintenance of stem cell homeostasis in the SAM. The solid black lines represent direct interactions, while the dashed black lines indicate that direct interactions have not been reported. Arrows indicate promotion/promoting effects, and T-bars indicate inhibition/inhibiting effects. Blue arrows represent phosphorylation, and purple arrows represent dephosphorylation.

CLV1, a receptor for CLV3, is crucial for its signal transduction (Figure 2). *CLV1* encodes a leucine-rich repeat-receptor-like kinase (LRR-RLK) [57], which is expressed mainly in the CZ. Its extracellular LRR domain can bind to CLV3 and form homodimers, after which the signal is transmitted into the cell to ultimately restrict the expression of *WUS* [58,59]. Loss-of-function *CLV1* mutants exhibit enlarged inflorescence meristems and an increased number of carpels [57].

Although *CLV2* also contains an LRR domain similar to that of *CLV1*, it neither binds to *CLV3* nor possesses an intracellular domain; thus, it cannot transduce signals independently [60]. It is expressed broadly not only in the SAM but also in many other tissues [31]. Compared with the *clv1* and *clv2* single mutants, the *clv1 clv2* double mutant presents a larger SAM and a greater number of carpels, similar to the *clv3* mutant [61]. These findings indicate that *CLV2* plays a role independent of *CLV1* in the regulation of SAM homeostasis [31]. The CRN plays an important role in the functional activity of CLV2. *CRN* encodes a receptor-like cytoplasmic kinase (RLCK) that lacks an extracellular domain [62]. *crn* can enhance the meristem-defective phenotype of *clv1* but not that of *clv2* [32]. While CRN alone resides in the endoplasmic reticulum, its interaction with CLV2 facilitates plasma membrane localization and the formation of an active heterodimeric receptor. This complex, which relies on the intrinsic kinase activity of the CRN rather than its adapter function, jointly transduces the CLV3 signal [60,63,64,65].

Another LRR receptor kinase, RPK2, is also involved in CLV3 perception. The *rpk2* mutant presents an enlarged shoot apical meristem and a reduced sensitivity to exogenous CLV3 peptide treatment. Notably, the extracellular LRR domain of RPK2 fails to bind CLV3 [58]. RPK2 is predominantly expressed in the PZ, exhibiting a pattern distinct from and non-overlapping with that of CLV1. Despite its structural similarity to CLV1, RPK2 does not interact with CLV1/CLV2 complexes, suggesting that it functions within a parallel pathway to negatively regulate the stem cell population and maintain SAM homeostasis [33].

The CLV1 homolog BARELY ANY MERISTEM 1 (BAM1) also serves as a direct receptor for CLV3. BAM family members (BAM1, BAM2, and BAM3) play redundant roles in maintaining SAM size, as evidenced by the reduced SAM size of the *bam1 bam2 bam3* triple mutant. Beyond CLV3 perception, BAM1, expressed in the PZ, also binds the CLE40 peptide. This BAM1-CLE40 module acts antagonistically to the CLV3 pathway by upregulating *WUS* expression in the OC, thereby promoting stem cell proliferation, which is in turn negatively regulated by WUS through a feedback mechanism that represses CLE40 [58,66]. Interestingly, compared with *clv1* single mutants, *clv1 bam1 bam2* and *clv1 bam1 bam2 bam3* mutants exhibit a larger SAM, indicating that *BAMs* also function redundantly with *CLV1* to inhibit SAM size [67,68,69]. CLV1 transcriptionally inhibits *BAM* genes, whose expression domains are non-overlapping. Specifically, *BAM1* and *BAM2* are expressed primarily in the PZ, whereas CLV1 is confined to the CZ. BAM3 is absent from the SAM but is detected in newly initiated primordia and vascular bundles. Notably, in *clv3* and *clv1* mutants, BAM1 and BAM3 exhibit ectopic expression in the RM, a region that overlaps with the normal expression domain of CLV1. This suggests that in the absence of *CLV1*, *BAM1* and *BAM3* can be ectopically localized to the region where *CLV1* is normally expressed, thereby partially compensating for *CLV1* function [69,70]. Therefore, the homeostasis of stem cells in the SAM is maintained through two interconnected pathways: in the CZ, the CLV3-CLV1-WUS signaling pathway inhibits WUS activity to regulate the number of stem cells; in the PZ, the CLE40-BAM1-WUS signaling pathway regulates cell differentiation [66].

### 4.2. The Coreceptor CIK: A Phosphorylation-Dependent Regulator of Meristem Homeostasis

As coreceptors for CLV1, CLV2, and RPK2, CIKs are LRR-RLKs that are broadly expressed across the SAM and play essential roles in its homeostasis (Figure 2). Genetic analyses demonstrated that CIK1-CIK4 functions redundantly in this process: the *cik1/2/3/4* quadruple mutant exhibits a dramatically enlarged and flattened SAM, with expanded expression domains and elevated transcript levels of *CLV3* and *WUS*, resembling those of *clv* mutants. Quintuple mutants combining *cik1/2/3/4* with *clv1*, *clv2*, or *rpk2* show meristem defects indistinguishable from those of the *cik* quadruple mutant, whereas the *wus cik1/2/3/4* quintuple phenocopies *wus* meristem termination, placing CIKs genetically within the same pathway as the known CLV receptors and upstream of WUS [34]. At the protein level, CIKs interact with CLV1, CLV2, and RPK2 both in vivo and in vitro and are directly phosphorylated by CLV1. Furthermore, CIK phosphorylation is rapidly induced upon CLV3 peptide treatment in a CLV1/BAM receptor-dependent manner. Phosphorylation at sites S456, T459, S601, S602, and S604 is essential for CIK3 function, establishing CIKs as critical signaling components that integrate multiple CLV receptor inputs to regulate stem cell homeostasis through posttranslational modification [34].

### 4.3. CLV1-PBL-POL Phosphorylation Cascade via Shoot Apical Meristem Homeostasis

The intricate regulation of CLV1 extends beyond transcriptional control to posttranslational mechanisms involving phosphorylation. Upon binding of the CLV3 peptide to its extracellular domain, CLV1 is activated through intermolecular autophosphorylation, a process that is abolished by site-directed mutagenesis of a conserved lysine residue (K720E) [71,72]. The phosphorylation status of CLV1 is dynamically regulated within the receptor complex. While CLV3 binding induces serine autophosphorylation of CLV1, the presence of other coreceptors, such as CLV2/CRN and RPK2, attenuates its phosphorylation level, suggesting their role in fine-tuning CLV1 kinase activity [65,71]. Following signal initiation, CLV1 is dephosphorylated to terminate signaling. This is mediated by kinase-associated protein phosphatase (KAPP), which is widely expressed throughout the SAM and encompasses the restricted expression domain of CLV1 in the OC [57,71]. KAPP preferentially binds to the phosphorylated form of CLV1, and their interaction forms a phosphorylation-dependent negative feedback loop: CLV1 autophosphorylation enhances KAPP binding, and CLV1 further transphosphorylates KAPP, which in turn dephosphorylates CLV1 to attenuate signaling. Consistent with this model, KAPP overexpression phenocopies *clv1* mutants, underscoring its critical role in modulating CLV1-mediated signaling homeostasis [71,72,73].

Following the perception of CLV3 by cell surface receptors, the signal is relayed into the cytoplasm through a group of receptor-like cytoplasmic kinases, namely, PBS1-LIKE 34/35/36 (PBL34/35/36) (Figure 2). These kinases are broadly expressed in the CZ, PZ, and OC, overlapping with *CLV1* expression [19,74]. The *pbl34 pbl35 pbl36* triple mutant displays a slightly increased SAM size and, more notably, an increased carpel number in flowers, indicating progressive disruption of meristem regulation [19]. Consistent with these findings, the expression domains of *CLV3* phenocopies *wus* and *WUS* are expanded, and the *CLV3* transcript is significantly upregulated, suggesting that PBL34/35/36 acts as downstream mediators in the CLV3-WUS pathway. At the molecular level, PBL34/35/36 physically interacts with multiple CLV pathway receptors, including CLV1, BAM1/3, and CIKs, and is phosphorylated by CLV1 and BAM1. PBL34 and BAM3 engage in mutual phosphorylation, which is essential for their function, as evidenced by the loss-of-function L135F mutation, which impairs both autophosphorylation and transphosphorylation activities [19,74]. Thus, PBL34/35/36 serve as critical cytoplasmic signaling nodes, transmitting extracellular CLV3 and related CLE peptide signals from membrane receptors to intracellular targets, ultimately contributing to the precise control of stem cell homeostasis.

In addition to KAPP, two protein phosphatases, POLTERGEIST (POL) and POL-LIKE1 (PLL1), also participate in the CLV1 pathway (Figure 2). *POL* is expressed throughout the meristem, whereas *PLL1* expression is confined to the OC [75,76]. Genetic evidence firmly places POL/PLL1 downstream of CLV signaling: *pol* single mutants are phenotypically wild-type but partially suppress meristem defects in strong *clv1* and *clv3* mutants and nearly fully suppress weak *clv1* mutants [77]. The *pol pll1* double mutant phenocopies the *wus* mutant, producing flowers with reduced organ number, and the *clv3 pol pll1* triple mutant is indistinguishable from *pol pll1*, demonstrating complete epistasis of *pol pll1* over *clv3* [78,79].

Mechanistically, PBL34/35/36 relay signals from CLV1/BAM receptors to POL/PLL1 via phosphorylation. PBL34 phosphorylates the tandem S-X-X-L motifs in POL’s N-terminus, which negatively regulates POL function. Phosphomimetic POL shows reduced interaction with the BAM3 intracellular domain, fails to rescue developmental defects in *pol pll1* mutants, and does not affect carpel number in the *clv1 pol* background, whereas wild-type and nonphosphomimetic POL fully complement these phenotypes. In the absence of CLE peptide ligands, POL and PLL associate with the CLV receptor complex at the plasma membrane, dampening receptor kinase activity and suppressing downstream signaling to prevent inappropriate pathway activation. Upon the perception of CLE peptides, activated receptors phosphorylate PBLs, which in turn phosphorylate POL/PLL. This phosphorylation event promotes the dissociation of POL/PLL from the receptor complex, thereby alleviating the suppression of signaling. Consequently, the activated pathway can then transmit signals to the nucleus to repress stem cell-promoting genes, ultimately inhibiting the overproliferation of stem cells in shoot and floral meristems [19,74] (Figure 2).

### 4.4. MAPK Phosphorylation Cascade in the Regulation of Shoot Apical Meristem Homeostasis

MAPK cascades have been implicated as potential intracellular transducers of the CLV3 signaling pathway (Figure 2). The exogenous CLV3 peptide (CLV3p) rapidly and transiently activates MPK3 and MPK6 phosphorylation in the SAM, primarily through the CLV1 and BAM1 receptors. Loss of MPK3/6 function in conditional double mutants results in increased SAM expression, expanded WUS and CLV3 expression domains, and insensitivity to CLV3p-induced SAM termination, demonstrating the necessity of these genes for stem cell restriction. CLV receptors differentially regulate MAPK activity: CLV1 acts as a CLV3-dependent negative regulator of MPK6, whereas RPK2 and CLV2/CRN positively modulate its activation. When co-expressed, these receptors counterbalance each other, collectively fine-tuning MAPK activity to maintain signaling homeostasis. Thus, the MAPK cascade serves as a critical intracellular bridge, transducing extracellular CLV3 signals into phosphorylation-mediated repression of WUS expression and stem cell proliferation, enabling spatially precise control across SAM zones and dynamic niche maintenance [65,80].

### 4.5. Spatial Control of the Stem Cell Niche by the ERf/EPFL Signaling Module

In addition to the canonical CLV3 signaling pathway, ERECTA family (ERf) receptors play an indispensable role in maintaining SAM homeostasis, contributing to the delicate balance between stem cell proliferation and organ initiation. This family comprises three functionally redundant members—*ER*, *ERL1*, and *ERL2*—such that only the triple mutant exhibits a flattened, widened SAM with a substantially enlarged stem cell domain [70,71] (Figure 2). This phenotype resembles that of a weakened CLV3-WUS feedback loop, suggesting that ERf signaling normally restricts the radial expansion of the stem cell niche.

The ERf pathway is activated at the SAM periphery by four EPFL ligands—EPFL1, EPFL2, EPFL4, and EPFL6—which are expressed in boundary regions. The functional significance of these ligands is demonstrated by the *epfl* quadruple mutant, which phenocopies the enlarged SAM observed in the *er erl1 erl2* triple mutant [72]. A core function of the ERf/EPFL module is to spatially confine the expression domains of the key stem cell regulators *WUS* and *CLV3* to the CZ of the SAM; in the *er erl1 erl2* mutant, the expression of both genes expands laterally into the peripheral zone [73,74,75].

Further analyses have revealed that CLV3 primarily regulates the cellular concentration of *WUS* within the CZ, whereas ERf signaling predominantly restricts the spatial domain of *WUS* expression, preventing its encroachment into the periphery. These two regulatory modes are functionally complementary and largely independent [81]. Based on these findings, an updated model for SAM regulation has been proposed: CLV3 acts in an autocrine manner to modulate *WUS* levels in the CZ, while ERfs provide paracrine signals from the meristem boundaries to restrict the radial expression domains of both *WUS* and *CLV3*. Together, they ensure the spatial integrity and quantitative stability of the stem cell niche [81]. This boundary-derived paracrine signaling effectively complements the canonical CLV3-WUS autocrine feedback loop, highlighting the importance of neighboring cell-derived peptide signals in maintaining meristem homeostasis [82].

Although the function of ERECTA as a protein kinase in other plant developmental processes is relatively well understood, its direct phosphorylation substrates in the context of SAM homeostasis remain to be elucidated [76]. Collectively, the ERf/EPFL pathway acts from the periphery of the SAM to radially confine the stem cell domain by repressing ectopic expression of *WUS* and *CLV3*, while simultaneously coordinating stem cell behavior across distinct tissue layers, thereby ensuring proper meristem function and development.

### 4.6. Receptor Kinases as Regulators of Meristem Homeostasis in Crops

The CLV signaling pathway, which is essential for meristem homeostasis in Arabidopsis, is functionally conserved but notably diverse in cereal crops such as maize. In maize, multiple orthologs of the Arabidopsis CLV components have been identified, each of which plays distinct but overlapping roles in regulating meristem development and influencing agronomically important traits. Maize *THICK TASSEL DWARF1* (*TD1*) encodes an LRR-RLK, which is considered to be a homolog of the Arabidopsis CLV1 protein. TD1 not only functions in vegetative development but also plays a role in restricting meristem size in inflorescences. Compared with the wild-type plants, the *td1* mutants are slightly shorter, and their floral meristems, as well as male and female inflorescences, are also affected [83]. Variations in FASCIATED EAR2 (FEA2), the homolog of CLV2 in maize, result in increases in inflorescence meristem size and kernel row number (Taguchi et al., 2001 [84], Je et al., 2016 [49]). Mutation of ZmCRN, the homolog of CRN, also leads to the enlargement of the SAM in maize. ZmFEA2 interacts with ZmCRN to cooperatively perceive the signal of ZmFCP1, while ZmFEA2 also interacts with CT2 (Gα) to mediate the signal of ZmCLE7, revealing how diverse signaling peptides can activate different downstream pathways through common receptor proteins [85]. In summary, the CLV pathway in maize is orchestrated by a network of receptors, including TD1, FEA2, FEA3, and ZmCRN. This network demonstrates both the conservation of core meristem size regulation and functional diversification, allowing for the perception of multiple peptide signals. The integration of these signals through shared components such as FEA2 enables precise control over stem cell proliferation, ultimately shaping inflorescence architecture and determining key yield traits. This elaborate understanding of maize CLV signaling provides valuable insights for crop improvement.

The core CLV signaling pathway also exhibits remarkable functional conservation in tomato. *FASCIATED AND BRANCHED* (*FAB*) encodes the homolog of CLV1, whereas *SlCLV2* encodes the homolog of CLV2, and mutations in either gene enlarge the SAM in tomato [51,86]. *FAB* is broadly expressed across various tissues, including the meristem. Its primary function is to act as the main signaling receptor for the arabinosylated CLV3 peptide. Upon binding its ligand, the FAB/CLV1 complex initiates an intracellular signaling cascade that ultimately restricts the expression domain of the stem cell-promoting transcription factor *WUS*. Loss-of-function mutations in *FAB* result in a moderate increase in the SAM and FM. This manifests phenotypically as weakly fasciated flowers with increased organ numbers, branched inflorescences, and fruits with a greater number of locules than the wild type does [51]. CRISPR-Cas9-engineered null mutants of *SICLV2* exhibit a nearly indistinguishable phenotype from that of *fab* mutants, characterized by weak fasciation, increased floral organ number, and a moderate increase in fruit locule number. The similarity of their loss-of-function phenotypes indicates that their functions overlap significantly in controlling meristem size [51,86]. In summary, genetic evidence from tomato strongly indicates that the CLV1/CLV2 receptor module—comprising FAB and SlCLV2—is functionally conserved and plays a critical, overlapping role in maintaining meristem homeostasis. This conserved mechanism ensures proper organogenesis and directly affects fundamental agronomic traits, such as fruit morphology and yield architecture. Moreover, the arabinosyltransferase STP2 mediates the arabinosylation of the SlCLV3 peptide under low-temperature conditions, a post-translational modification that is essential for its maturation and bioactivity, thereby ensuring normal fruit development under fluctuating environmental conditions [87]. In tomato, SlKNU directly represses both the stem cell marker gene *SlCLV3* and the receptor gene *SlCLV1* to regulate floral meristem determinacy. Notably, CRISPR/Cas9-generated SlKNU loss-of-function mutants exhibit increased fruit size, underscoring the potential of this regulatory node for yield improvement [88].

Similarly, in broccoli, the MAPK cascade within the SAM serves as a core component of the thermoresponsive transcriptional module during head formation. Upstream LRR-RLKs, including ERECTA, function as hub proteins that orchestrate the growth–defense trade-off under heat stress by coordinating MAPK signaling with phytohormone networks, thereby balancing stress acclimation and reproductive development [89].

The CLV–WUS regulatory module, governed by reversible protein phosphorylation, is functionally conserved across diverse crop species including maize, tomato, and broccoli, where it directly shapes inflorescence architecture, fruit size, and stress resilience. Harnessing this conserved phosphorylation signaling network through precision genome editing and molecular breeding holds tremendous potential for enhancing yield-related traits and developing climate-resilient crop varieties.

Comparative analysis of SAM homeostasis regulation in Arabidopsis, maize, and tomato reveals that the CLV3–WUS module plays a central role across species. Systematic comparison of CLV signaling components demonstrates that this pathway exhibits both remarkable functional conservation and significant divergence. Loss-of-function mutations in CLV pathway genes consistently result in SAM enlargement and increased organ numbers, with certain traits conferring potential agronomic benefits, while WUS orthologs are universally required for stem cell maintenance across species [25,29,49,51]. Despite the highly conserved core regulatory framework, notable differences exist between Arabidopsis and crops. In Arabidopsis, CLV3 is primarily perceived by CLV1 as the main receptor. In maize, however, TD1 (the CLV1 ortholog) and FEA2 (the CLV2 ortholog) exhibit more complex genetic interactions, with ZmCRN, the CRN ortholog, participating in the regulation of inflorescence meristem size [83,85]. Tomato shows further functional diversification of receptor components, where FAB, as the CLV1 ortholog, plays a predominant role in regulating carpel number, whereas SlCLV2 functions in partial redundancy with FAB [51,86]. In addition, ERECTA-family kinases in broccoli coordinate thermoresponsive developmental processes under heat stress [89]. These cross-species comparisons not only reinforce the central role of phosphorylation-mediated CLV signaling in SAM homeostasis but also reveal how phosphorylation outputs are modulated by species-specific receptor diversification and ligand–receptor interaction dynamics. The observed similarities and differences caution against the direct extrapolation of molecular models established in Arabidopsis to crop species, emphasizing the necessity of systematic analyses that take into account the unique genetic networks and developmental contexts of each species.

## 5. Perspective

The maintenance of SAM homeostasis relies on a coordinated network of small peptides, receptor kinases, and intracellular signaling. Central to this process are receptor kinases, which perceive extracellular signals and transduce them intracellularly. This signal transduction, which is critically mediated by phosphorylation, plays a key role in regulating SAM homeostasis and cell differentiation.

Although the crucial CLV3-WUS regulatory pathway has been established [26] and the immediate phosphorylation events following receptor activation from CLV1 to the cytoplasmic kinase PBL and subsequently to the phosphatase POL are being delineated, the complete signaling cascade remains fragmented. The identification of several receptor kinases in the pathway has not been matched by a clear understanding of their phosphorylation substrates or regulatory mechanisms. A key gap lies in the phosphorylation-dependent regulation of CLV1 itself. Although CLV1 is known to autophosphorylate and be dephosphorylated by KAPP [71,72], its functional phosphorylation sites remain unreported. While CLV1 transmits its signal by phosphorylating the cytoplasmic kinase PBL, the specific phosphorylation sites on PBL have yet to be identified. Furthermore, although PBL subsequently phosphorylates the phosphatase POL to propagate the signal, the counteracting phosphatase that dephosphorylates PBL to antagonize CLV1 activity remains unknown.

There remains a need to address unresolved questions regarding the downstream phosphorylation substrates and regulatory mechanisms of receptor kinases. Beyond phosphorylation, a key signaling question remains: how does CLV1, through downstream kinases such as PBL and MPK, ultimately regulate the transcription factor that controls *WUS* expression? To systematically elucidate the downstream phosphorylation substrates, regulatory mechanisms, and signaling network of receptor kinases (such as CLV1) involved in the regulation of stem cell homeostasis, a comprehensive experimental strategy can be implemented. First, phosphoproteomic profiling using LC-MS/MS combined with IMAC/TiO_2_ enrichment will be employed to dynamically identify functional phosphorylation sites on key kinases (e.g., CLV1 and PBL) and their potential substrates, capturing time-resolved modification patterns during SAM development. Next, interaction mapping and validation should be conducted. Protein-protein interactions and kinase—substrate relationships will be mapped using coimmunoprecipitation (Co-IP) and proximity labeling. In vitro and in vivo kinase assays will subsequently be performed to functionally validate critical phosphorylation events. Finally, functional genetics will be pursued through a three-pronged CRISPR strategy: (i) conditional knockouts of core components using tissue-specific drivers to circumvent embryonic lethality; (ii) endogenous base editing at the identified phosphosite (Ser-to-Ala for phospho-dead; Ser-to-Asp for phospho-mimetic), coupled with a *pWUS:erGFP* reporter to quantify the spatial and temporal dynamics of *WUS* expression in the SAM; and (iii) promoter-swap complementation (WUSpro vs. CLV3pro) to distinguish whether the phosphorylation switch acts cell-autonomously in the organizing center or non-cell-autonomously in stem cells via feedback regulation. This framework will establish whether the phosphorylation event serves as a permissive rheostat or an instructive binary switch for WUS-dependent stem cell homeostasis.

Studying the molecular basis of SAM regulatory mechanisms, when integrated with the aforementioned strategies, will overcome the limitations of existing knowledge and facilitate in-depth development in the fields of botany and biotechnology. An in-depth understanding of these mechanisms will provide a significant theoretical basis and technical support for the breeding of new varieties of high-yield crops.

## Data Availability

No new data were created or analyzed in this study.
